# Questionable assumptions mar modelling of Kenya home‐based testing campaigns

**DOI:** 10.1002/jia2.25230

**Published:** 2019-01-22

**Authors:** Reuben Granich, Somya Gupta, Brian G Williams

**Affiliations:** ^1^ Independent Public Health Consultant San Francisco California USA; ^2^ Independent Public Health Consultant Delhi India; ^3^ South African Centre for Epidemiological Modelling and Analysis Stellenbosch South Africa

**Keywords:** HIV, AIDS, HIV treatment, treatment as prevention, epidemiologic model, resource needs, multi‐disease prevention campaign

## Competing interests

The authors have no competing interests to declare.

## Authors’ contributions

RG/SG/BW drafted, read and reviewed final version of letter.

Dear Editor,

We read your recent article *Optimal timing of HIV home‐based counselling and testing rounds in Western Kenya* 1 with considerable interest. The authors make questionable assumptions about their modelled reference campaign in which 90% of the population is reached, of these 60% of those contacted are linked to care, and of these only 75% receive the CD4 count test required to access ART. Of people on ART, 86% are virally suppressed, and of these 95.5% are retained at one year and only 88% after four years. Therefore, the proportion of people living with HIV (PLHIV) in community who are virally suppressed for the first year can be derived by multiplying the parameters: diagnosed (90%), linked (60%), lab tested (75%), virally suppressed (86%) and retained at one year (95.5%). Using these parameters, the denominator rapidly decreases and the percentage of HIV‐positive people that are virally suppressed after a year is about 30% and it declines thereafter. Using these parameters results in an ineffective and inefficient treatment programme at a substantial cost. The article then asks what the optimal timing would be if it were to be repeated? Given the poorly performing programme, one answer is that it should first be significantly improved before considering the question of how often it should be repeated.

There are many examples of well‐performing programmes and successful interventions. For example, the 2008 private‐public sector collaboration involving the Kenya Ministry of Health, Centers for Disease Control, CHF International, and Vestergaard Frandsen showed that an effective community‐based multi‐disease prevention campaign in Western Kenya was able to reach and test 87% of its target population over a seven days period 2. By offering long‐lasting insecticide‐treated bed nets, water filters and rapid HIV testing they reached 47,311 people with a 96% uptake of the multi‐disease prevention package with 99.7% accepting HIV testing including 18,101 (38%) men. Of participants, 80% had never been tested and 4% were diagnosed with HIV. The campaign also reached people with higher CD4 cell counts probably earlier in their HIV disease 2, 3. The more recent SEARCH study in Uganda and Kenya has also reached very high proportions of the community with testing of 131,307 (89%) of and successful treatment with minimal loss to follow‐up 4, 5. These programmes and recent results from population‐based incidence studies offer further evidence that expanding access to well‐designed and implemented HIV services can achieve remarkable impact 6, 7, 8, 9, 10.

On closer examination the model in the article examines the utility of repeating a failing home‐based counselling and testing (HBCT) programme when compared to more successful models. HBCT programmes that reach 90% of its intended target are expected, but a programme that can only put 60% on treatment clearly has major problems that require urgent redress. The subsequent 55% losses due to unnecessary waits for CD4 cell counts and other service delivery problems further reduce the overall percentage on treatment and suppressed. Perhaps most worrisome about the modelled programme is the assumption that no one who is lost to follow‐up returns.

Despite considerable and effort to reach people in their homes, the combined result is that the model projects that only around 30% of PLHIV are successfully diagnosed, treated and virally suppressed. Additionally, the cost of treatment is $367 which is very expensive given the poor performance and the current annual costs of around $75 per year for ARVs alone. In many programmes these avoidable high costs and low performance measures would be a sign of failure and indicate the need for significant revision in service delivery to prevent avoidable illnesses, deaths and HIV infections. Specifically, interventions to improve performance that other successful programmes have used include same‐day treatment without delays for unnecessary laboratories, immediate social support for patients starting treatment, routine use of viral load for monitoring success and motivating patients, a highly tolerable dolutegravir‐based regimen, and the use of cohort monitoring with field support to ensure that people who are lost do not stay so permanently.

It is important to build models using past performance and costs but, in this case, it appears that the proposed programme would need to be significantly revised or perhaps even stopped until proper policies and quality assurance systems can be put in place. The questionable parameterization leaves the reader in the dark regarding the potential benefits of a well‐functioning programme combined with multiple multi‐disease prevention campaigns. The projected health impact and economic savings of this sort of frontloaded approach are considerable and have been discussed elsewhere 3, 11. It is likely that diagnosing and offering sustained successful treatment to most of the people living in the community would effectively extinguish the epidemic. There would be a need for long‐term treatment to keep PLHIV healthy and non‐infectious. With most people on treatment the programme could then focus efforts on active case management, reaching partners with HIV self‐tests and services, and other targeted prevention efforts for the rare HIV transmissions in the community. The value of effectively reaching 95‐95‐95, HIV elimination and ending AIDS 12 in the community is difficult to state with certainty but is important to consider. Science and experience support modelling focused on determining the impact of well‐functioning programmes that rapidly scale access to proven HIV services including early access to high levels HIV treatment and viral suppression.

Sincerely,

Reuben Granich

Somya Gupta

Brian Williams

Geneva, Switzerland
